# Exploring the Role of Portable Ophthalmic Devices in Clinical and Nonclinical Settings: Qualitative Study of Ophthalmologists and Optometrists

**DOI:** 10.2196/87040

**Published:** 2026-10-05

**Authors:** Jeremy Barton, Sarika Gopalakrishnan, Eric Singman, Michael E Rogers, Donald Fletcher

**Affiliations:** 1 Envision Research Institute, Envision Inc., 610 N. Main Street, Wichita, KS, 67203, United States, 1 3164401509; 2 Department of Physics Adjunct Instructor, Wichita State University, Wichita State University, Wichita, KS, United States; 3 Ophthalmology and Visual Sciences, University of Maryland, Baltimore, Baltimore, MD, United States; 4 Exercise Science, Professor, Wichita State University, Wichita, KS, United States; 5 Ophthalmologist and Medical Director, Envision Vision Rehabilitation, Wichita, KS, United States; 6 Smith-Kettlewell Eye Research Institute, San Francisco, CA, United States

**Keywords:** portable ophthalmic devices, handheld fundus camera, portable slit lamp, teleophthalmology, digital health, accessibility of eye care, vision screening, ophthalmic imaging, eye care delivery, clinician perceptions, qualitative research

## Abstract

**Background:**

Access to eye care is a persistent challenge due to the high global burden of visual impairment and barriers to receiving eye care, such as transportation and cost. Conventional ophthalmic equipment is immobile and unsuitable for bedside or community use, and this limits timely diagnosis and care delivery. Portable ophthalmic devices, such as handheld slit-lamps and fundus cameras, offer the potential to extend diagnostic capabilities to nonclinical settings and improve accessibility of eye care.

**Objective:**

This study examined eye care professionals’ perceptions and experiences with portable ophthalmic devices, focusing on their roles in both clinical and nonclinical settings.

**Methods:**

Thirty-one practicing eye care professionals (16 ophthalmologists, 15 optometrists; 16 female, 15 male) with 2 to 45 years of clinical experience (mean 20.5, SD 13.1 y) participated. Semistructured interviews were conducted in person or via videoconferencing. The interview included open-ended qualitative questions on current and past use, benefits, and desired features of portable devices, along with quantitative ratings of device attributes on a 1 to 10 scale. Transcripts were coded in NVivo using thematic analysis, and descriptive statistics were calculated for the quantitative data.

**Results:**

Participants described portable devices as essential in settings where conventional equipment is impractical, including hospital rooms, emergency departments, nursing homes, and outreach. They emphasized utility for patients with mobility challenges and cognitive impairments. Key benefits included earlier disease detection and support for screening and referrals. Challenges involved image quality and device stabilization. Quantitative ratings of portable device attributes showed the highest scores for speed (median 8, IQR 6-8) and ease of use (median 8, IQR 6-8).

**Conclusions:**

Portable ophthalmic devices expand access to eye care beyond standard clinics and accommodate patients underserved by traditional equipment. Addressing technical and usability challenges and enhancing training opportunities will strengthen their integration into routine and outreach care.

## Introduction

Visual impairment and vision loss remain a major global health concern. As of 2019, at least 2.2 billion people globally were visually impaired, with about 1 billion of those cases being preventable or unresolved. While uncorrected refractive errors account for much of this burden, ocular trauma, glaucoma, and other conditions also contribute significantly [1,2]. Early access to eye care has consistently been shown to improve visual outcomes, particularly following ocular trauma [3,4].

In the United States, accessibility of eye care concerns often stems from medical costs and accessibility issues [5,6]. The economic burden of vision impairment in the United States is substantial. Recent estimates suggest that vision loss and blindness cost approximately US $134.2 billion annually, including nearly US $99 billion in direct costs to the health care system for medical services [1]. At the individual level, people living with vision impairment face significantly higher annual health care expenditures, averaging nearly US $17,000 more per person compared to those without vision loss.

One of the largest factors for accessibility is the lack of transportation options to visit an eye care clinic [7,8]. Transportation challenges may prevent individuals who are at risk of ocular disease from attending routine examinations or screenings. Difficulty acquiring transportation can lead to delayed diagnoses and reduced opportunities for timely intervention, thereby contributing to preventable vision loss and exacerbating disparities in ocular health outcomes [5,6].

Additionally, reaching an eye care professional is not only made difficult by transportation issues but also by a global shortage of trained eye care service providers in some regions [1,2]. Global estimates indicate there are fewer than 250,000 ophthalmologists worldwide, with low-income countries averaging fewer than 4 ophthalmologists per million people and high-income countries averaging just over 76 per million [9]. This shortage limits specialist availability and contributes to delayed medical intervention.

These accessibility barriers are further compounded by a reliance on traditional ophthalmic equipment, which is typically large, immobile, and costly to operate [5,10]. These ophthalmic systems require dedicated space, making them impractical in nontraditional or resource-limited settings. As a result, patients in rural areas often go without appropriate screening or diagnosis, and existing ophthalmology workforces remain overextended. Additionally, for patients with limited mobility, these larger ophthalmic devices can pose challenges, sometimes requiring additional modifications to the device to be accessible [11-13].

Portable ophthalmic devices have emerged as a potential solution to bridge these gaps. Handheld fundus cameras and compact slit-lamps enable eye assessments to be conducted outside of conventional clinic environments, thereby extending diagnostic capacity into community and primary care settings. Earlier iterations of these technologies were limited by image quality or lack of digital integration; however, more recent devices have demonstrated sufficient quality for screening diabetic retinopathy and other ocular conditions [14-18]. Their handheld nature has also helped in addressing issues with accommodating older adults with mobility problems [19].

Prior work has emphasized technical performance metrics, such as image quality or diagnostic accuracy; however, less is known about the practical integration of these devices into everyday workflows. Previous studies have focused on device accuracy in specific clinical contexts [12,20,21], and there exists comparatively less literature about how eye care professionals perceive the benefits and challenges of portable ophthalmic technologies. Understanding these perspectives is critical for informing device development, integration with telemedicine, and training strategies for both specialists and non–eye care professionals. This study addresses this key gap in the literature by moving beyond device validation to explore how portable ophthalmic technologies are perceived and applied by clinicians. These perceptions are key to generating actionable insights that can inform device design and ultimately advance accessibility to eye care.

## Methods

### Ethical Considerations

This study employed in-depth qualitative interviews to capture in-depth insights of perceptions toward portable ophthalmic devices and a brief qualitative assessment of measurable trends in device attributes. This research was conducted at the Envision Research Institute in Wichita, Kansas, with ethical approval granted by the Wichita State University Institutional Review Board (study number 5737), in accordance with the principles of the Declaration of Helsinki. All participants provided informed written consent prior to participation and were asked to reaffirm verbally upon the start of the interview that they had consented to participating. Participants were deidentified with a randomized participation ID.

### Participant Recruitment

A total of 31 participants were interviewed. This sample included 16 ophthalmologists and 15 optometrists. These professionals had a range of clinical experience from 2 to 45 years, with a mean of 20.5 (SD 13.1) years. Several participants also had prior experience practicing in other countries. A sample of 31 participants exceeds the typical thresholds recommended for achieving thematic saturation in qualitative health research [22,23]. The point at which no new themes emerged was monitored throughout data acquisition and analysis to confirm thematic saturation.

Participants were recruited from professional networks affiliated with the Envision Vision Rehabilitation Center and Envision University. Participants were also recruited from the primary researcher’s networking at the Envision conference and the American Academy of Ophthalmology conference. Participants were contacted via emails that they had shared previously within these networks or with the primary researcher. Eligible participants included currently practicing ophthalmologists or optometrists within the United States. To mitigate sampling bias, recruitment was extended to clinicians practicing in a range of clinical environments and geographic locations. Both ophthalmologists and optometrists were included to capture diversity in professional perspective and scope of practice. Inclusion criteria required that participants be actively engaged in eye care practice and have had some prior experience with portable ophthalmic devices. Individuals were contacted via email and/or phone and were provided with a recruitment flyer detailing the study purpose and participation criteria. Interested participants contacted the primary researcher by phone and/or email and scheduled time to be interviewed.

### Materials

A semistructured interview guide (Multimedia Appendix 1) was developed to explore clinician perspectives on the use and potential of portable ophthalmic devices. The interview guide’s design was shaped by existing literature about portable diagnostic technologies and accessibility challenges in eye care. The research team consulted with several practicing ophthalmologists and optometrists during the early design phase to help ensure clinical relevance in the interview guide.

The interview guide was designed to capture detailed qualitative data about portable ophthalmic devices from eye care professionals. The guide contained open-ended questions about current and past use of portable ophthalmic devices, their perception of their benefits, and the features or functions that portable devices needed to be beneficial. Participants were also asked about situations in which they found portable devices necessary and whether they anticipated purchasing additional devices in the future. Additionally, participants were asked about the feasibility of training non–eye care professionals to operate these devices for basic imaging and screening.

Lastly, the guide included rating scale questions about portable ophthalmic device attributes. These questions contained attributes such as importance, usability, ease of use, efficiency, and affordability on a 1 to 10 scale, where 1 represented a poor score and 10 represented an excellent score. Participants were encouraged to elaborate on their numerical ratings to provide context. This combination of open-ended and structured items enabled both a rich thematic analysis of professional perspectives and a complementary quantitative comparison of how particular attributes were valued.

### Procedure

Interviews were conducted between August 2024 and April 2025 via videoconferencing software: Microsoft Teams or Zoom. Occasionally, in-person interviews were arranged when it was more convenient for the participant. During in-person interviews, participants spoke into a microphone that was connected to teleconference software to enable transcription to be done the same as when interviews were conducted remotely. Video was disabled during all interviews so that only audio was recorded via the teleconferencing software’s recording function. The interviewer is visually impaired, so lack of nonverbal cues was present across all interviews. Each session lasted approximately 20 minutes. The interviewer used follow-up probes when clarification or elaboration was needed. Interviews were transcribed using the built-in transcription functions of Zoom or Teams. A researcher listened to the recorded audio and verified each transcript. Transcripts were then edited in Microsoft Word when disparities between the captured audio and transcription were found. Any identifiable information was removed from transcripts during the verifying process.

### Data Analysis

Verified transcripts were uploaded into NVivo (version 15.0; Lumivero) for systematic qualitative coding and analysis. A thematic analysis approach was used because the study was exploratory and intended to identify patterns in clinician perceptions. Transcripts were reviewed line by line, where text segments were highlighted and assigned an inductive code based on key language or repeated ideas. For example, mentions of “wheelchair” or “hospital” were initially grouped as subcodes based on the clinical or patient contexts described. These first-order subcodes were grouped into a hierarchical structure comprising themes, subthemes, codes, and subcodes. Thematic saturation was monitored throughout data collection, and no new codes were identified after approximately 21 interviews, suggesting adequate coverage.

After preliminary coding was completed by the primary researcher, 2 additional researchers independently reviewed the coding structure to reduce bias. Discrepancies were resolved through collaborative discussion and voting until a consensus was reached for each code. This process ensured intercoder reliability and strengthened the thematic structure. The finalized codebook was exported into Microsoft Excel and organized to facilitate visualization of the coding hierarchy and frequency distributions across participants. This Excel spreadsheet was referenced to create thematic hierarchy figures within Microsoft Visio.

For the rating scale questions, participants’ responses were manually input and analyzed in Microsoft Excel. Descriptive statistics, reported as median (IQR), were calculated for each rated attribute of portable ophthalmic devices. The quantitative ratings provided a numerical structure complement to the qualitative findings by offering a way to compare the relative importance of portable device attributes, such as speed and quality, across participants.

Multiple strategies were employed to enhance the rigor and credibility of the qualitative data. These included consensus of coding from 3 independent investigators during coding, literature review of the thematic structure, and transparency in reporting the coding hierarchy. The thematic hierarchy was iteratively reviewed against the data throughout the entire coding process. The combination of qualitative and rating-scale attribute data provided a comprehensive understanding of the applications, benefits, and implementation challenges of portable ophthalmic technologies as perceived by eye care professionals.

## Results

### Participant Characteristics

A total of 31 eye care professionals were interviewed for this study, comprising 16 ophthalmologists and 15 optometrists. The sample included 16 female and 15 male participants. Participants had a wide range of professional experience, spanning from 2 to 45 years in the field, with a mean of 20.5 (SD 13.1) years of practice. These participants provided insights into the use, benefits, and limitations of portable ophthalmic devices across various care settings, patient populations, and clinical contexts (Word Cloud: Multimedia Appendix 2).

### Thematic Analysis

#### Theme 1: Current and Past Experience With Portable Ophthalmic Devices

All participants had direct, hands-on experience using portable ophthalmic devices, ranging from handheld slit lamps and fundus cameras to portable tonometers and indirect ophthalmoscopes. Participants used these tools routinely during hospital consults and in community screening events. Participants also cited using these devices to examine specific patient populations such as the immobile or homebound.

*I do have a portable slit lamp that I use primarily for people who are wheelchair bound. And when I have to make house calls, for people who can’t get out, who are immobile.*

[P012]

A number of participants noted using devices adapted for digital imaging or smartphone compatibility, and several had tested or piloted portable technologies in clinical or research settings. Even among those not currently using these tools, there was broad awareness of their availability and potential.

*I have experience with a portable fundus camera that can be actually mounted on an iPhone.*

[P20]

All participants have used a nondigital portable ophthalmic device for viewing, but only 29% (9/31) of participants have used a handheld imaging device with digital capabilities in a professional capacity. All participants were aware of handheld digital fundus cameras and other similar devices but had not yet incorporated these devices into their professional setups. In total, 29% (9/31) of participants expressed any current interest in purchasing portable handheld devices in the future.

*The idea of being able to do in-home evaluations for some seniors with vision loss could be a useful tool. But now it’s not something that’s on a priority list for me, but I do see the benefit for it.*

[P011]

#### Theme 2: Features and Functions

##### Clinician Perspectives on Device Features and Functionality

Participants provided perspectives on the features and functions of portable ophthalmic devices, both on the strengths of current technologies and areas requiring improvement. As illustrated in the thematic framework (Figure 1), these insights clustered around subthemes of desired functionality and perceived flaws.

**Figure 1.**
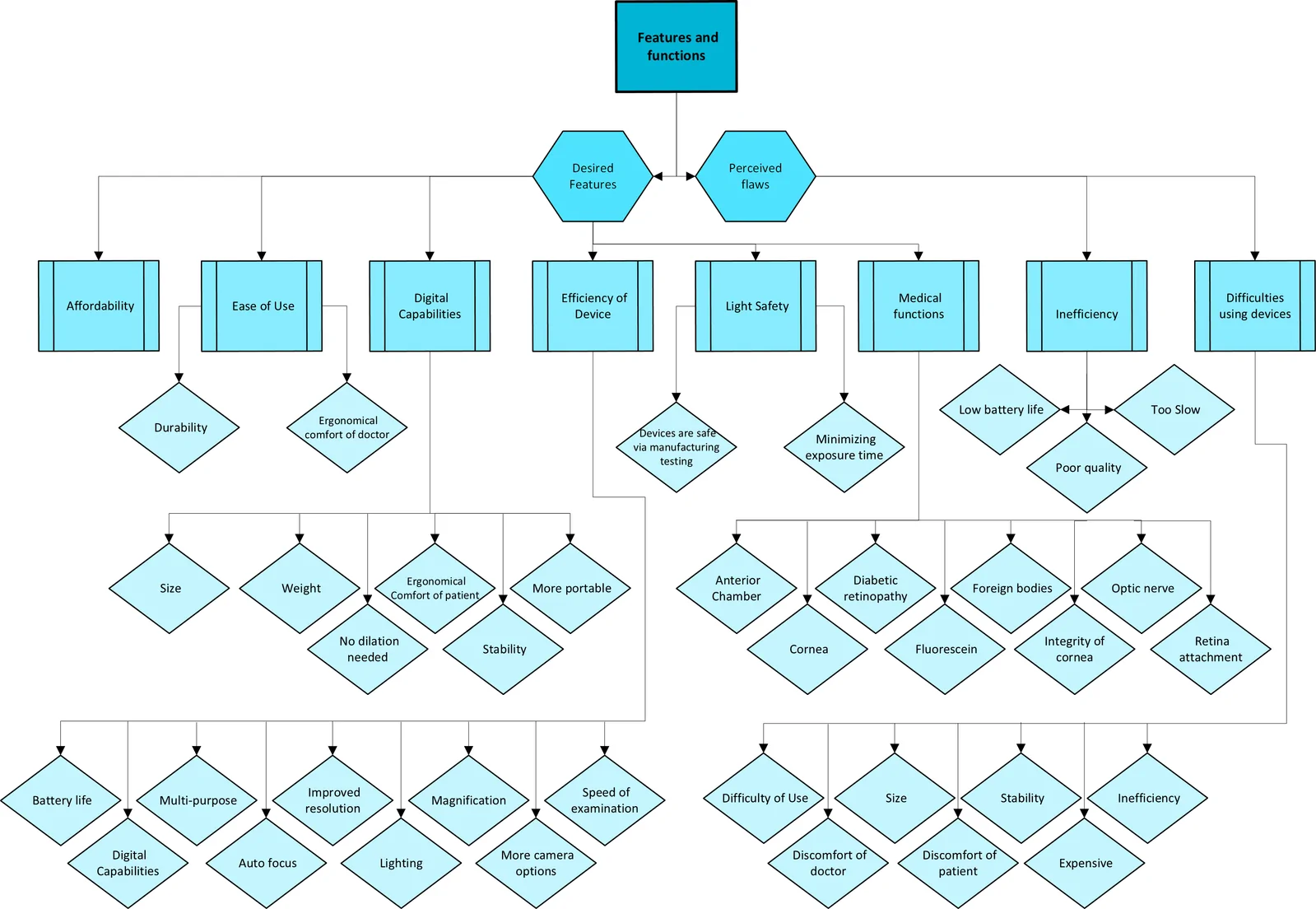
Thematic framework of features and functions. This flowchart outlines the hierarchical structure of the thematic analysis of the theme features and functions. It illustrates what features were most desirable to participants and what functions of current devices were perceived as flawed.

##### Perceived Concerns With Using Handheld Devices

Participants raised several recurring concerns regarding the limitations of current functionality of portable ophthalmic devices. One of the most frequently mentioned issues was image quality, particularly when compared to tabletop versions.

*I really don't think they are as good as the in office slit lamp.*

[P024]

Participants noted that it was difficult to capture clear, diagnostic-quality images without proper patient stabilization. The absence of chin rests or headrests in many portable models made it challenging to examine physically limited patients or those with behavioral characteristics that make it difficult to sit still. They cited that this concern was elevated in outreach or bedside settings where there were no surfaces to rest or stabilize the device. This issue of stabilization was frequently referenced as a point of frustration, and for some participants, it even was the main barrier to why they would not adopt it into their own practices.

*I really think that those portable slit lamps have to have a chin rest or some sort of head stabilization unit that the portable slit lamp fits in. And the reason I say that is because, if you don't stabilize a chin rest, it’s very difficult to get a clear in focus picture every single time.*

[P021]

Clinicians also highlighted their own discomfort while using handheld devices during examinations, citing poor ergonomics and weight distribution as barriers to prolonged examinations or screening efforts. Participants shared that the battery life and fragility of certain models limited their utility in rural or mobile contexts. A few participants expressed that while they saw the potential in digital imaging features, they found the software or data transfer systems difficult to use or poorly integrated with clinical workflows.

##### Desired Functions and Improvements

Participants shared a clear vision for what functions they believe portable ophthalmic devices should have or be integrated into future devices. Participants wanted devices that are durable and easy to operate in nonclinical settings. The ability to capture high-quality anterior and posterior segment images in a single unit was frequently mentioned as an ideal feature.

*I would love something that is able to capture both anterior segment and posterior segment photos consistently and with clarity in the image.*

[P003]

Respondents also expressed strong support for devices with digital capabilities, including high-resolution imaging, wireless data transmission, and compatibility with medical data secure storage systems. Features like autofocus and automated image capture were identified as ways to reduce the need for patient cooperation and improve ease of use.

*An auto capture of the image would be good as well, because it would ensure that you have the best quality of the image that you can get with that device on that person in that moment.*

[P020]

Several participants stressed the importance of including standard diagnostic tools, such as fluorescein capability, within portable formats. Cost was also a recurring theme; devices must be affordable enough to justify intermittent use, especially in outreach or low-resource settings.

### Theme 3: Role of Portable Ophthalmic Devices

#### Extending Eye Care Beyond Traditional Settings

Participants emphasized the role of portable ophthalmic devices across a wide range of settings and situations. The thematic framework in Figure 2 shows that these devices were consistently described as tools that expand access to care and enable eye examinations in environments where conventional equipment is unavailable or impractical.

**Figure 2.**
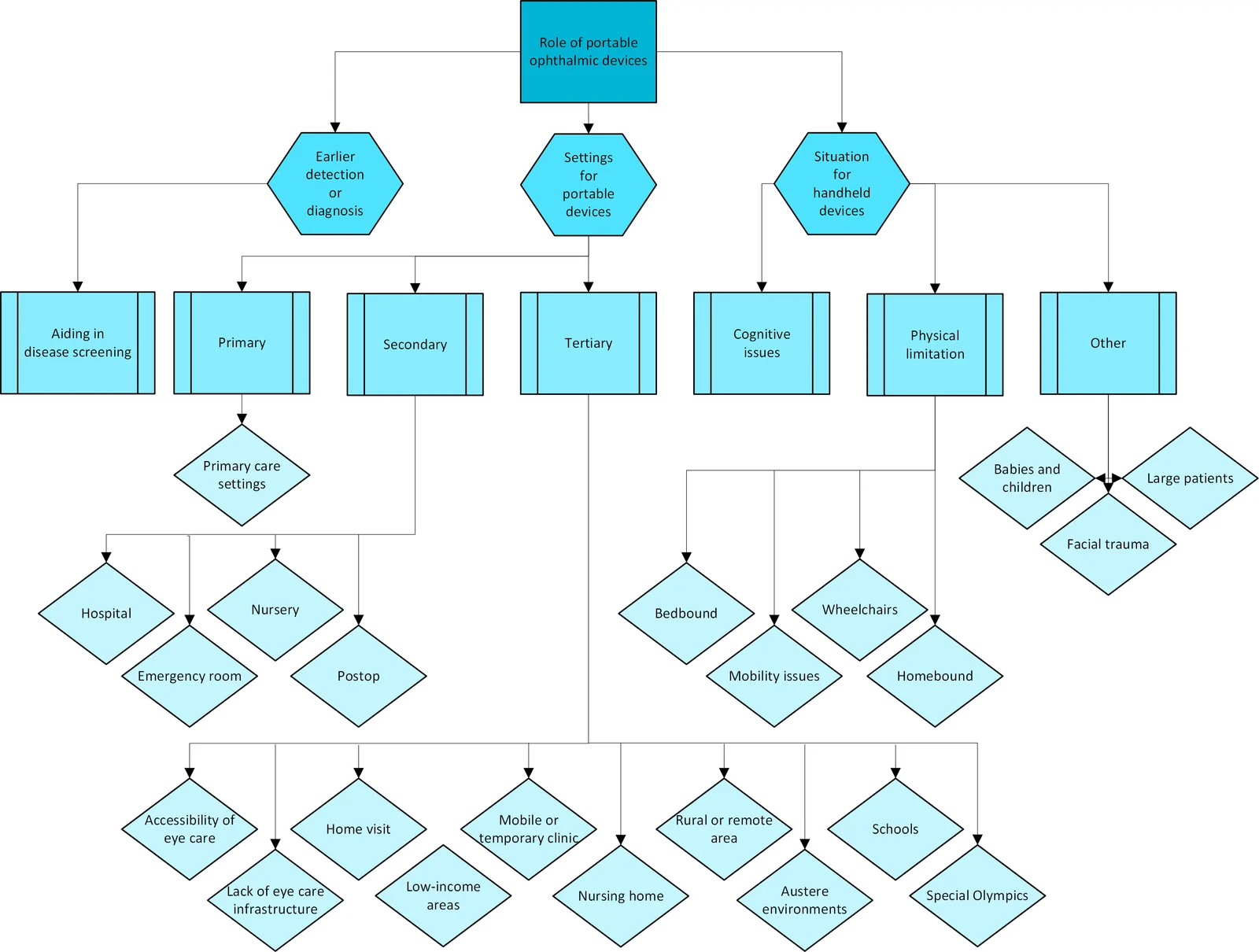
Thematic framework of role of portable ophthalmic devices. This flowchart outlines the hierarchical structure of the thematic analysis of the theme role of portable ophthalmic devices. It illustrates the perceived benefits portable ophthalmic devices had in certain situations and settings.

#### Early Detection of Conditions and Screening

Thematic analysis illuminated participants’ perception of the critical role portable ophthalmic devices play in eye care. As mentioned in the introduction, earlier detection of diseases is critical for preventing blindness. Portable devices have the advantage of being transportable, allowing eye care professionals to increase the accessibility of eye care to aid in diagnosing eye issues earlier.

*Getting a good quality picture of the back of the eye, we can catch things before it turns into a possibly blinding situation. Because looking at the back of the eye, we can catch potential strokes, and we can catch undiagnosed diabetes.*

[P006]

Doctors also underlined these devices’ roles in earlier detection of diseases and screening. The convenience of handheld ophthalmic devices enables them to be used in mobile clinics to screen for diseases, such as diabetic retinopathy.

*And just screenings, you would be surprised by the things you find in the back of somebody’s eye, even if they don’t have any symptoms yet. So, we can catch things earlier.*

[P006]

#### Perceived Beneficial Settings for Portable Ophthalmic Devices

Participants provided insights into what settings portable ophthalmic devices are perceived as most useful. Participants emphasized that these tools are vital in scenarios of outreach and mobile checkups. Their portability allows them to be used in remote or rural areas where eye care infrastructure may be lacking.

*When it comes to healthcare, accessibility is the biggest issue of our time. So, we need to be able to get to people who can’t get to us.*

[P019]

One of the more mentioned nonclinical settings was long-term care facilities, such as nursing homes or assisted living (Table 1). The demographic of patients present in these facilities often have mobility or transportation issues that prevent them from traveling to a clinic.

*When I’m working with patients who are immobile, who can’t be positioned at a slit lamp. Often times in a nursing home, a lot of my patients are advanced Alzheimer’s. Portable ophthalmic devices can help with cooperation and getting them in the correct position.*

[P013]

These facilities are unlikely to have ophthalmic equipment, and participants cite needing to bring as much portable equipment as is feasible to these locations to provide comparable care to a typical clinical setting.

**Table 1.** Most frequently mentioned settings and patient situations for portable device use (N=31).

Categories and description	Values, n (%)
Settings	
Hospital	13 (42)
Rural or remote area	13 (42)
Nursing home	11 (35)
Mobile or temporary clinic	8 (26)
Emergency room	6 (19)
Situations	
Wheelchairs	11 (35)
Babies and children	7 (23)
Cognitive issues	6 (19)
Facial trauma	4 (13)
Bedbound	5 (16)

#### Perceived Beneficial Situations for Portable Ophthalmic Devices

Participants additionally provided detailed insights into specific patient situations where portable devices are critical. These situations could accompany the settings stated earlier, but these situations were also present in typical eye care professionals’ clinical setups. Most of the time, participants cited that these situations stem from tabletop devices requiring “a level of physical and mental aptitude” (P009), which excludes many vulnerable patients from receiving proper exams.

Nineteen participants mentioned that when they have patients with mobility issues, such as patients in wheelchairs or who were bedbound, using handheld devices is critical to accommodate them (Table 1). Doctors emphasized that patients in wheelchairs are sometimes not able to reach their clinic’s typical slit lamp or fundus camera.

*I do have a portable slit lamp that I use primarily for people who are wheelchair bound.*

[P012]

Participants stated that portable devices are also useful to allow patients who can’t keep their head in a slit lamp due to facial trauma. Additionally, 6 doctors shared the opinion that it is easier to accommodate those with cognitive impairments with handheld devices versus larger countertop versions. The smaller nature of handheld devices was observed to cause less anxiety in patients and to require less cooperation compared to countertop versions.

*You hold it and they look at it and it’s a lot less scary, especially for patients with mental disability. They just have to look at the light and sit there. They don’t have to put their head in a scary thing that looks like it’s going to poke you in the eye.*

[P006]

### Theme 4: Role of Telemedicine in Ophthalmology

#### Perceived Benefits and Limitations of Telemedicine

When discussing portable handheld devices with digital capabilities, participants also provided a great deal of insight into their opinions on telemedicine in ophthalmology and training non–eye care professionals to take diagnostic-quality photos that can be sent to specialists (Figure 3). Participants commented on how virtual visits with patients only served a smaller role, such as diagnosis of conjunctivitis or dry eye, and were not suitable as a replacement for an in-person exam for most of the types of patients they were seeing. One participant expressed that they do not find telemedicine as critical to the field of ophthalmology.

*I’m not convinced that telemedicine in ophthalmology will be that useful. First of all, the patients are not going to have these (portable ophthalmic devices) at home right. If I’m going to go there where they are. They might as well come to me, right?*

[P013]

Most participants reiterated many strengths of telemedicine, while still emphasizing that they are not always a suitable replacement for in-person examinations. The overwhelming benefit, described by these physicians, was the role it plays in aiding in referrals. Digital images help in tracking patient history, but they all were viewed to streamline the referral process by potentially having specialists see images of the patient’s eye before the patient arrives at the clinic.

*I think the benefits would be an increase in teamwork across specialties from your PCP (Primary care physician). You could send the picture to an ophthalmologist, to an optometrist or to a low vision specialist, so that you can see exactly what’s going on between referrals. So, everybody’s on the same page.*

[P006]

**Figure 3.**
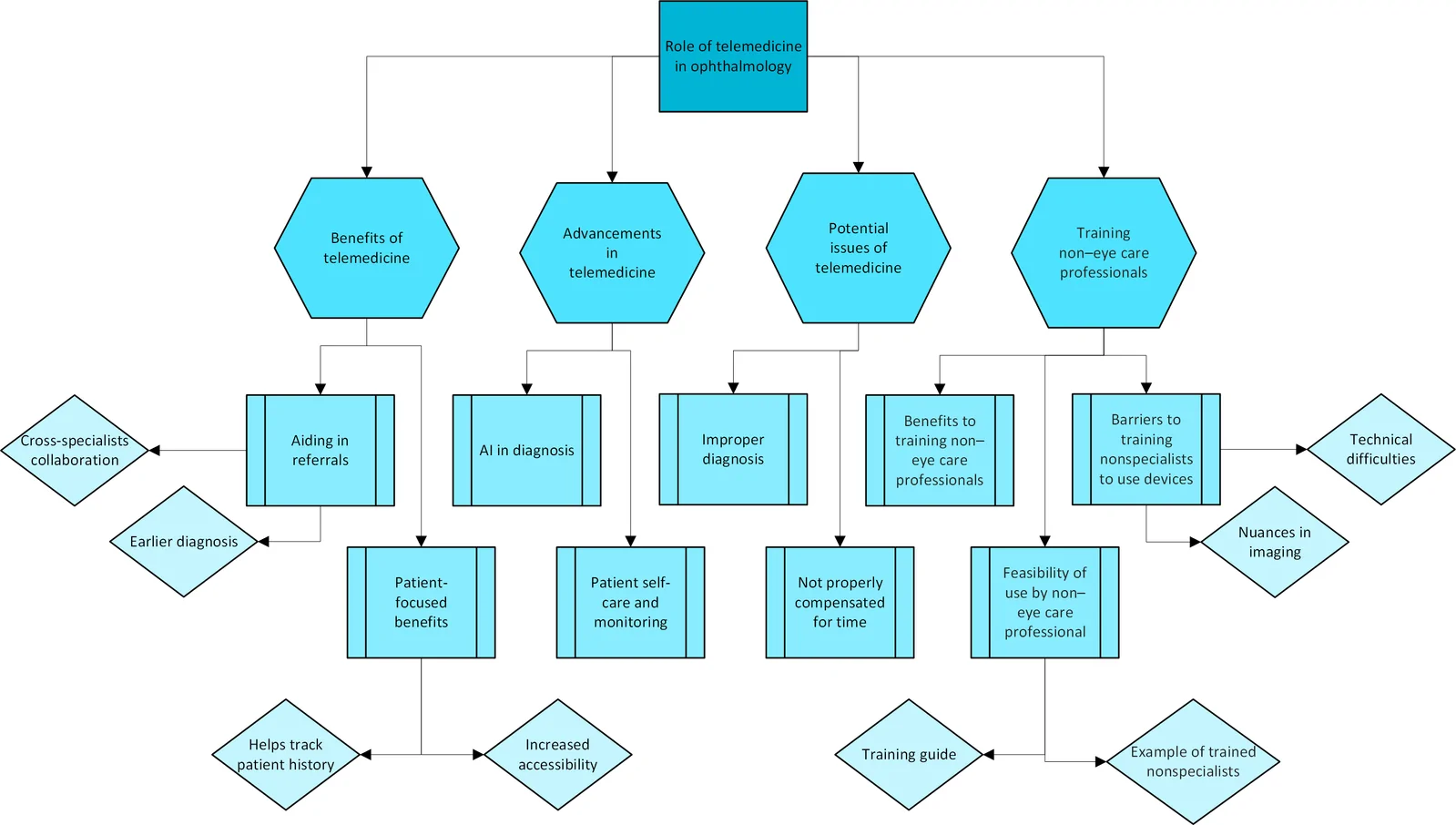
Thematic framework of role of telemedicine in ophthalmology. This flowchart outlines the hierarchical structure of the thematic analysis of the theme role of portable ophthalmic devices. It illustrates what role telemedicine was perceived to have in ophthalmology and what improvements can be made to its integration into existing health care systems.

#### Advancements in Telemedicine

Participants expressed specific advancements in technology that were perceived as beneficial to teleophthalmology and digital portable ophthalmic devices. One participant expressed the need for integration of AI aiding in referrals by being able to analyze images and recommend specialists.

*I think there is a big need to develop portable technology capable, with AI, to check patients for diabetic retinopathy with smaller portable retinal cameras.*

[P014]

Participants also conveyed a need for the technology of smartphones to advance enough that it would be possible for patients to take pictures of their eyes at home, with sufficient quality to be useful for diagnostic purposes. This would enable patients to self-monitor at home and send images to the appropriate specialists.

*At my clinic sometimes on weekends, or as an emergency, patients take a picture, and they think that it’s clear enough to make a diagnostic and they request some help as. If we can develop technology capable of getting good images at home. At least we could advise this patient to come as soon as possible.*

[P014]

#### Training Non–Eye Care Professionals

Many of the perceived benefits of portable ophthalmic devices, particularly those with digital capabilities, require taking photos of patients when an eye care professional is not present. When questioned about the feasibility of training non–eye care professionals to take photos of eyes, participants provided insight into the need for such training to increase accessibility of eye care, but also into the barriers and difficulties non–eye care professionals would encounter.

*I have heard, of telemedicine type projects, where the big barrier was getting the staff in the general doctor’s office to feel comfortable obtaining images for the ophthalmology setting.*

[P018]

Eye care professionals expressed concerns about the difficulties that non–eye care professionals could encounter in being trained to take reliable images with these types of devices. Participants stressed that ideally, the device should be as easy to use as possible, but also that any personnel be trained ahead of time before being required to take images of a patient.

*It’s not easy, It’s not. But I think it is necessary.*

[P021]

### Ratings on Portable Ophthalmic Device Attributes

Participants were asked to rate their perceptions of attributes of portable ophthalmic devices they had used prior to this study. Responses were treated as interval-level data on a 1 to 10 scale, and scores were summarized by calculating the median (IQR). Speed and ease of use each received the highest ratings, with a median score of 8 (IQR 6-8; Table 2). Comfort (IQR 6-8), efficiency (IQR 6-8), quality (IQR 5-8), and importance (IQR 4-9) in current practice each received a median score of 7, while affordability received the lowest median rating of 6 (IQR 5‐7). Overall, these ratings show that participants generally viewed portable ophthalmic devices favorably, particularly with regard to workflow-related characteristics such as speed, ease of use, comfort, and efficiency. Quality also received a positive median rating of 7 (IQR 5‐8), but its broader distribution aligns with qualitative findings that image quality remains a commonly cited limitation of current portable technologies.

The widest IQRs were observed for importance (4-9) and affordability (5-7). These findings suggest greater variation in how participants perceived the overall value of portable ophthalmic devices compared with their functional performance. Differences in clinical settings likely contributed to this variation. Participants working in hospitals, nursing homes, outreach programs, and other nontraditional care environments frequently described portable devices as essential tools, whereas those practicing primarily in conventional clinic settings often viewed them as less critical. One participant summarized this contrast by stating, “If I’m going to go to the nursing home, they are a 10; in my office they are a 0” (P013). Collectively, these findings indicate that perceptions of device value are strongly influenced by the clinical context in which portable technologies are used.

**Table 2.** Participant ratings of portable device attributes on a 1 to 10 scale.

Device attribute	Values, median (IQR)
Speed	8 (6-8)
Ease of use	8 (6-8)
Comfort	7 (6-8)
Efficiency	7 (6-8)
Quality	7 (5-8)
Importance	7 (4-9)
Affordability	6 (5-7)

### Summary

Eye care professionals perceived portable ophthalmic devices as important tools for expanding access to care, particularly in nontraditional settings and among patients with mobility or cognitive limitations. Participants highlighted their value for screening, early disease detection, and facilitating referrals through telemedicine, while also identifying image quality and device stabilization as key limitations. Quantitative findings demonstrated favorable perceptions of workflow-related attributes such as speed and ease of use, although opinions regarding the overall importance and value of these devices varied across practice settings.

## Discussion

### Principal Findings

This study explored clinician perspectives on portable ophthalmic devices, highlighting their perceived benefits and opportunities for broader integration into eye care delivery. Unlike prior studies, which have largely focused on device validation for specific disease screening in narrow clinic settings, this work draws on the experiences of 31 practicing ophthalmologists and optometrists representing a wide range of subspecialties and practice environments [24,25]. By combining qualitative thematic analysis with quantitative attribute ratings, this study provides a comprehensive account of how portable ophthalmic devices are perceived in real-world practice. It extends the literature beyond technical performance by capturing how these tools influence patient-centered care. The findings of this study underscore the unrealized potential of portable technologies in advancing equitable access to eye care across diverse populations and patient care settings.

Participants reported substantial hands-on experience with portable ophthalmic devices, particularly in managing patients with mobility limitations. This aligns with earlier reports of handheld slit lamps and fundus cameras improving access in nontraditional situations [16-18]. But when discussing their experience with digital portable devices, clinicians noted that while they were broadly aware of smartphone-adapted devices and digital fundus cameras, adoption into daily workflows remained limited. Specifically, only 29% (9/31) of participants mentioned that they had prior experience with these devices in their own clinical setups, and only 29% (9/31) of participants expressed interest in purchasing these devices in the future. This disconnect suggests implementation barriers that warrant further investigation. Without deliberate strategies to address these gaps, patients most in need of these tools, such as those with mobility issues, may remain underserved.

Image quality and device stabilization are frequently cited functions of portable devices in need of improvement. This is consistent with prior device validation studies of handheld fundus cameras and slit-lamp adapters [21,26]. However, these clinician perspectives add an important distinction between limitations that are image resolution–based and other issues that directly affect providers’ willingness to adopt devices in routine practice. If a handheld fundus camera requires awkward positioning or frequent retakes, the workflow burden could outweigh potential benefits. This highlights a difference between device performance in validation studies and usability in real-world clinical settings. Bridging this gap may require iterative design informed by direct clinician feedback, as well as rigorous evaluation of workflow integration and long-term adoption. Specifically, clinician feedback in this study would suggest that future devices should be designed with some sort of stabilization accessory in mind. Similarly, this focus is being shown in studies about integrating teleophthalmology more heavily into eye care, where usability and interoperability are increasingly prioritized alongside clinical accuracy [27,28].

The discussion of digital device features emphasized this ongoing shift in expectations for device functionality. Integrated imaging and data-sharing capabilities were viewed as key to facilitating telemedicine-enabled referrals. These insights align with prior work demonstrating the importance of portable imaging tools in enabling earlier detection of ocular disease and accelerating referral pathways [27,28].

A central implication of these findings is that the role of portable devices is not to replicate conventional tabletop equipment but instead to extend care into contexts where standard tools fail. This included reaching more remote areas without access to eye care clinics and addressing accessibility issues in typical clinical settings, such as treating patients who are in wheelchairs or pediatric patients who struggle to use traditional countertop equipment. These reported perceptions align with previous publications about reducing disparities in eye care [12-14,20]; however, the strong emphasis on wheelchair users and cognitively impaired patients underscores an accessibility gap that is often underacknowledged in ophthalmic device usage. This demonstrates that further integration of these devices into clinics should be considered by physicians to best address these accessibility concerns.

Findings in this study related to telemedicine extend this perception of the capability of portable devices to increase accessibility of eye care. The importance of digital image capture for facilitating triage and referrals was consistently emphasized; however, the concern professionals have about the image quality of portable devices being sufficient for proper clinical diagnosis was also highlighted. This complements evidence that teleophthalmology programs, particularly in diabetic retinopathy screening, depend on high-quality portable imaging to function effectively [24,28]. However, eye care professionals were clear in presenting telemedicine as a complement rather than a substitute for in-person examination. Eye care professionals highlighted that while telemedicine is critical to expanding care to those who cannot come into a clinic, clinicians still preferred to examine patients in person if they are able. There was also enthusiasm expressed for future devices to incorporate AI and at-home imaging capabilities to aid in facilitating faster triage and referrals. To that end, however, to realize this potential, standards for secure data sharing and legal responsibility must be clarified.

The potential role of non–eye care professionals in operating portable devices was discussed alongside telemedicine as a means to integrate portable devices into currently existing health care systems. Participants recognized that enabling nurses and allied health workers to conduct basic screening could extend access in underserved areas. Such approaches mirror successful models in diabetic retinopathy screening [29,30]. Yet concerns were raised regarding ease of training and image quality control, underscoring the importance of user-friendly interfaces and guided feedback mechanisms. Inexperienced users may produce images that are not diagnostically useful, limiting the value of screening programs. These findings point to a critical opportunity for developers of portable ophthalmic devices to design devices that support usability and training instructions to quickly onboard new operators.

It was continually emphasized that the importance of these devices is highly context-dependent, as shown by the high variance in importance and cost ratings in this study. A tool viewed as indispensable in a nursing home may be deemed unnecessary in a well-equipped clinic. This suggests that while these devices are shown to be beneficial, implementation strategies should vary depending on the clinical environment and patient population. This should be considered by policymakers, who will need to evaluate cost-effectiveness across settings to guide rational deployment.

### Study Limitations

Participants drew upon their experiences with a variety of portable ophthalmic devices, rather than evaluating a single standardized technology. As a result, perceptions may reflect differences in device design and functionality, which could contribute to variability in responses, particularly within the quantitative ratings of device attributes. Additionally, participants were recruited through professional conferences and continuing education networks. Clinicians who engage in these activities may be more likely to adopt or express interest in emerging technologies, potentially introducing selection bias and resulting in more favorable perceptions of portable ophthalmic devices than may be present in the broader eye care community. The primary researcher has a visual impairment and conducted interviews without access to nonverbal cues. Although standardized interview procedures, transcript review, and investigator triangulation were employed to enhance rigor, the absence of visual cues may have influenced the interpretation of certain participant responses.

### Future Directions

Future research should aim to assess the real-world performance of specific portable ophthalmic devices in diverse practice environments. Comparative studies could help quantify the diagnostic accuracy and workflow efficiency of these tools relative to conventional equipment. There is also a clear need to develop and evaluate structured training curricula for non–eye care professionals, especially in the context of telemedicine and outreach care. Finally, future studies should explore cost-effectiveness, implementation challenges, and long-term patient outcomes associated with portable device use in low-resource settings.

### Conclusion

Portable ophthalmic devices represent a promising means to expand access and equity in eye care, particularly for underserved populations and patients with mobility or cognitive limitations. This study highlights that their greatest value lies not in replicating conventional tabletop instruments, but in enabling care delivery where traditional equipment is impractical or unavailable. By amplifying clinician reach, portable technologies have the potential to reduce inequities in eye care access and improve early detection of vision-threatening conditions. Improvements to policy and clinical practices should be made to better integrate these devices into existing systems to best leverage that potential.

From a device design standpoint, manufacturers should address persistent barriers of image quality and device stabilization, and prioritize ease of use to aid in device integration into health care systems. Wider adoption will depend on secure digital integration into teleophthalmology platforms and the development of structured training curricula that allow nonspecialist providers to participate safely in screening and imaging.

Future research should extend beyond validation of individual devices to assess their role in broader models of care. Comparative studies are needed to evaluate how portable technologies influence referral patterns and workflow efficiency. Implementation research should explore how portable devices can be deployed most effectively in rural and low-resource settings, and the economic burden of implementation.

Collectively, these directions point toward a future where portable ophthalmic devices are no longer seen as niche tools but as integral components of digitally connected eye care. By bridging technical innovation with thoughtful implementation, portable devices could help transform the landscape of ophthalmology into one that is more accessible, equitable, and resilient to the challenges of global vision health.

## Supplementary material

10.2196/87040 Multimedia Appendix 1 Interview guide and introduction script used during interviews of participants.

10.2196/87040 Multimedia Appendix 2 Word cloud representing the frequency that participants discussed each coded theme and subtheme.

## References

[1] (2019). World report on vision. https://iris.who.int/handle/10665/328717.

[2] Thylefors B (1992). Present challenges in the global prevention of blindness. Aust N Z J Ophthalmol.

[3] Perry M, Dancey A, Mireskandari K, Oakley P, Davies S, Cameron M (2005). Emergency care in facial trauma--a maxillofacial and ophthalmic perspective. Injury.

[4] Suwarno O (2003). Assessing and managing ophthalmic emergencies. JAAPA.

[5] Khan IA, Bashar MA, Tripathi A, Priyanka N (2024). The benefits and challenges of implementing teleophthalmology in low-resource settings: a systematic review. Cureus.

[6] Green N, Miller F, Khanna D (2024). Barriers to eye care for adults in the United States and solutions for it: a literature review. Cureus.

[7] Kim AB, Cheng BT, Tanna AP (2023). Delayed medical care due to lack of transportation among US adults with glaucoma. Ophthalmol Glaucoma.

[8] Hark LA, Radakrishnan A, Madhava M (2019). Awareness of ocular diagnosis, transportation means, and barriers to ophthalmology follow-up in the Philadelphia Telemedicine Glaucoma Detection and Follow-up Study. Soc Work Health Care.

[9] Resnikoff S, Lansingh VC, Washburn L (2020). Estimated number of ophthalmologists worldwide (International Council of Ophthalmology update): will we meet the needs?. Br J Ophthalmol.

[10] Kumar S, Tay-Kearney ML, Chaves F, Constable IJ, Yogesan K (2006). Remote ophthalmology services: cost comparison of telemedicine and alternative service delivery options. J Telemed Telecare.

[11] Javitt JC (1989). A modified slit lamp for examination of wheelchair-bound patients. Arch Ophthalmol.

[12] Haddock LJ, Kim DY, Mukai S (2013). Simple, inexpensive technique for high-quality smartphone fundus photography in human and animal eyes. J Ophthalmol.

[13] Ahn SJ, Kim YH (2024). Clinical applications and future directions of smartphone fundus imaging. Diagnostics (Basel).

[14] Labkovich M, Paul M, Kim E (2022). Portable hardware & software technologies for addressing ophthalmic health disparities: a systematic review. Digit Health.

[15] Sengupta S, Newman-Casey PA, Sindal M (2015). Screening for diabetic retinopathy with a portable non-mydriatic fundus camera. Invest Ophthalmol Vis Sci.

[16] Maamari RN, Keenan JD, Fletcher DA, Margolis TP (2014). A mobile phone-based retinal camera for portable wide field imaging. Br J Ophthalmol.

[17] Chalam KV, Chamchikh J, Gasparian S (2022). Optics and utility of low-cost smartphone-based portable digital fundus camera system for screening of retinal diseases. Diagnostics (Basel).

[18] Rajalakshmi R, Prathiba V, Arulmalar S, Usha M (2021). Review of retinal cameras for global coverage of diabetic retinopathy screening. Eye (Lond).

[19] Sakata R, Numaga J (2014). The utility of rebound tonometer in late elderly subjects. Clin Interv Aging.

[20] Fu L (2017). Use of smartphone funduscopy to aid diagnosis of chorioretinitis after donor–recipient mismatched cardiac transplantation. BMJ Case Rep.

[21] Russo A, Morescalchi F, Costagliola C, Delcassi L, Semeraro F (2015). Comparison of smartphone ophthalmoscopy with slit-lamp biomicroscopy for grading diabetic retinopathy. Am J Ophthalmol.

[22] Guest G, Bunce A, Johnson L (2006). How many interviews are enough? An experiment with data saturation and variability. Field methods.

[23] Squire CM, Giombi KC, Rupert DJ, Amoozegar J, Williams P (2024). Determining an appropriate sample size for qualitative interviews to achieve true and near code saturation: secondary analysis of data. J Med Internet Res.

[24] Tomić M, Vrabec R, Hendelja Đ, Kolarić V, Bulum T, Rahelić D (2023). Diagnostic accuracy of hand-held fundus camera and artificial intelligence in diabetic retinopathy screening. Biomedicines.

[25] Midena E, Zennaro L, Lapo C (2022). Handheld fundus camera for diabetic retinopathy screening: a comparison study with table-top fundus camera in real-life setting. J Clin Med.

[26] Collon S, Chang D, Tabin G, Hong K, Myung D, Thapa S (2020). Utility and feasibility of teleophthalmology using a smartphone-based ophthalmic camera in screening camps in Nepal. Asia Pac J Ophthalmol (Phila).

[27] Nguyen M, Fujioka J, Wentlandt K (2020). Using the technology acceptance model to explore health provider and administrator perceptions of the usefulness and ease of using technology in palliative care. BMC Palliat Care.

[28] Liu Y, Torres Diaz A, Benkert R (2019). Scaling up teleophthalmology for diabetic eye screening: opportunities for widespread implementation in the USA. Curr Diab Rep.

[29] Nguyen M, Stamenova V, Onabajo N (2022). Perceptions of a teleophthalmology screening program for diabetic retinopathy in adults with type 1 and type 2 diabetes in urban primary care settings. Can J Diabetes.

[30] Chen JS, Lin MC, Yiu G (2023). Barriers to implementation of teleretinal diabetic retinopathy screening programs across the University of California. Telemed J E Health.

