# Supplementary material for: Exploring the Role of Portable Ophthalmic Devices in Clinical and Nonclinical Settings: Qualitative Study of Ophthalmologists and Optometrists
Source: JMIR Med Inform. 2026 Oct 5;14:e87040. doi: 10.2196/87040 (PMC13637025; doi:10.2196/87040)
Supplement: Supplementary file 1 [file medinform-v14-e87040-s001.docx]

**Appendix 1: Interview guide**

**Introduction script**

Hello, my name is Jeremy and I represent Envision Research Institute as a Research Fellow. I appreciate your willingness to participate in my research. This study will examine the perception of eye-care professionals toward mobile/portable ophthalmic devices. You were selected for participation in this study based on your prior experience with mobile/portable ophthalmic devices.

Before we begin, I would like to share a few procedures for our conversation. You will be asked a series of questions about your experience with mobile/portable ophthalmic devices. These questions will pertain to assessing those devices necessity, efficiency, affordability and safety. Audio will be recorded, but not video, so please turn off your webcam before we begin (if applicable)

Although we will be on a first name basis, no names or identifying comments will be used when I report the results of this session. You can be assured of confidentiality.

This interview will last approximately 20 minutes.

Have you have read and signed the consent form?

Do you have any questions about it before we begin?

Is your webcam turned off?

Do I have your permission to audio record our interview?

| **Questions** | **Responses** |
| --- | --- |
| 1. **What mobile/portable ophthalmic devices do you have in your setup?**  1a.Have you used any in the past?  1b. Have you ever used any with digital capabilities? |  |
| **Questions about necessity and role** | |
| 2. **Do you have any specific mobile devices in mind to purchase in the future?**  2a.Are there any that you are aware of? |  |
| 3. **What situations are mobile ophthalmic devices needed in your setup?**  3a. Or what situations do you perceive them as being needed in other setups? |  |
| 4**. What specific functions do mobile ophthalmic devices serve in these situations?**  4a. You can be more technical in terms of what they need to be able to see/identify to be useful for diagnosis |  |
| 5. **What perceived benefits do you expect mobile ophthalmic devices to make in your setup?**  5a. What do you think these devices are meant to help with? |  |
| The next several questions will ask you for an answer on a 1-10 scale, but please feel free to take time to elaborate further on your answer if you want to. | |
| 6. **How important would you rate mobile ophthalmic devices in your setup?** 1-10 Not important to extremely important |  |
| **Questions about Usability** | |
| **Hardware** | |
| **7a. For the hardware controls of mobile ophthalmic devices, how would you rate the ease of the controls in general?** 1-10 Not easy to extremely easy |  |
| **7b. Does your answer change, and if so to what number, if we focus on the controls specific to the camera/optics/lighting? (moving a slit lamp, changing a lens etc.)** 1-10 Not easy to extremely easy |  |
| **7c. What about controls for powering or charging the device? (charging, batteries etc.) 1-10 Not easy to extremely easy** |  |
| 8a. **During an examination with the device, what level of comfort do you have while operating these devices?** 1-10 Not comfortable to extremely comfortable |  |
| 8b. **How comfortable do you think these devices are for patients?** 1-10 Not comfortable to extremely comfortable |  |
| **Software** (If applicable, meaning the device you have in mind has a digital display, if not put N/A) | |
| 9a. For mobile ophthalmic devices, how easy to understand is the user-interface in general? 1-10 Not easy to extremely easy |  |
| 9b. **Does your answer change, and if so to what number, if we focus on the user interface for data entry?** 1-10 Not easy to extremely easy |  |
| 9c.**What about the user interface for file transfer?** 1-10 Not easy to extremely easy |  |
| 9d. **What about the user interface for the camera?** 1-10 Not easy to extremely easy |  |
| **Questions about Efficiency** | |
| 10a. How would you rate the speed of the device? 1-10 Not satisfactory to extremely satisfactory |  |
| 10b. Quality of the device? 1-10 Not satisfactory to extremely satisfactory |  |
| 10c. Efficiency of the device? 1-10 Not satisfactory to extremely satisfactory |  |
| **Questions about affordability** | |
| 11. How do you rate the costs of these devices compared to their alternatives 1-10, extremely expensive to very affordable |  |
| **Questions about Safety** | |
| 12. How do you evaluate whether the light source of the mobile ophthalmic device is safe for your patient or not? |  |
| Summary/ending. Do you have any more thoughts or comments? Do you think it is feasible to train non-eye care professionals to use these devices.  Thank you for your time. |  |
