# Supplementary material for: Exploring the Role of Portable Ophthalmic Devices in Clinical and Nonclinical Settings: Qualitative Study of Ophthalmologists and Optometrists
Source: JMIR Med Inform. 2026 Oct 5;14:e87040. doi: 10.2196/87040 (PMC13637025; doi:10.2196/87040)
Supplement: Supplementary file 2 [file medinform-v14-e87040-s002.docx]

**Appendix 2: Word cloud and code frequency**

***Word cloud of coded references across themes and subthemes:*** A word cloud visualizing the frequency of coded references within the thematic framework. Larger terms indicate higher reference counts in NVivo, highlighting the most frequently discussed themes.
